# A novel copper-induced cell death-related lncRNA prognostic signature associated with immune infiltration and clinical value in gastric cancer

**DOI:** 10.1007/s00432-023-04916-7

**Published:** 2023-06-08

**Authors:** Li Wang, Ke Xiao, Zhaogang Dong, Tao Meng, Xiaowen Cheng, Yuanhong Xu

**Affiliations:** 1 Department of Clinical Laboratory, The First Affiliated Hospital of Anhui Medical University, Hefei, 230032 Anhui China; 2 Department of Clinical Laboratory, Qilu Hospital of Shandong University, 107 Wenhuaxi Road, Jinan, 250012 Shandong Province China

**Keywords:** Prognostic signature, Copper induced cell death, lncRNA, Gastric cancer, Biomarker

## Abstract

**Background:**

Gastric cancer (GC) is one of the most important malignancies and has a poor prognosis. Copper-induced cell death, recently termed cuproptosis, may directly affect the outcome of GC. Long noncoding RNAs (lncRNAs), possessing stable structures, can influence the prognosis of cancer and may serve as potential prognostic prediction factors for various cancers. However, the role of copper cell death-related lncRNAs (CRLs) in GC has not been thoroughly investigated. Here, we aim to elucidate the role of CRLs in predicting prognosis, diagnosis, and immunotherapy in GC patients.

**Methods:**

RNA expression data for 407 GC patients from The Cancer Genome Atlas (TCGA) were gathered, and differentially expressed CRLs were identified. Subsequently, the researchers applied univariate, LASSO, and multivariate Cox regression to construct a prognostic signature consisting of 5 lncRNAs based on the CRLs. Stratified by the median CRLSig risk score, Kaplan–Meier analysis was utilized to compare overall survival (OS) between the high- and low-risk groups. Among the two groups, gene set enrichment analysis (GSEA), tumor microenvironment (TME), drug sensitivity analysis, and immune checkpoint analysis were conducted. In addition, consensus clustering and nomogram analysis were performed to predict OS. Cell experiments and 112 human serum samples were employed to verify the effect of lncRNAs on GC. Furthermore, the diagnostic value of the CRLSig in the serum of GC patients was analyzed by the receiver operating characteristic (ROC) curve.

**Results:**

A prognostic signature for GC patients was constructed based on CRLs, composed of AC129926.1, AP002954.1, AC023511.1, LINC01537, and TMEM75. According to the K-M survival analysis, high-risk GC patients had a lower OS rate and progression-free survival rate than low-risk GC patients. Further support for the model’s accuracy was provided by ROC, principal component analysis, and the validation set. The area under the curve (AUC) of 0.772 for GC patients showed a better prognostic value than any other clinicopathological variable. Furthermore, immune infiltration analysis showed that the high-risk group had greater antitumor immune responses in the tumor microenvironment. In the high-risk subgroup, 23 immune checkpoint genes had significantly higher expression levels than in the low-risk subgroup (*p* < 0.05). The half-maximal inhibitory concentrations (IC50) of 86 drugs were found to be significantly different in the two groups. Accordingly, the model is capable of predicting the effectiveness of immunotherapy. In addition, the five CRLs in GC serum exhibited statistically significant expression levels. The AUC of this signature in GC serum was 0.894, with a 95% CI of 0.822–0.944. Moreover, lncRNA AC129926.1 was significantly overexpressed in GC cell lines and the serum of GC patients. Importantly, colony formation, wound healing, and transwell assays further confirmed the oncogenic role of AC129926.1 in GC.

**Conclusion:**

In this study, a prognostic signature model consisting of five CRLs was developed to improve OS prediction accuracy in GC patients. The model also has the potential to predict immune infiltration and immunotherapy effectiveness. Furthermore, the CRLSig might serve as a novel serum biomarker to differentiate GC patients from healthy individuals.

**Supplementary Information:**

The online version contains supplementary material available at 10.1007/s00432-023-04916-7.

## Introduction

With more than one million new cancer cases and 769,000 deaths, gastric cancer (GC) continues to be a major cancer worldwide and ranked fifth in incidence and fourth in mortality globally in 2020 (Sung et al. 2021). It has high incidence and mortality rates in East Asia, Eastern Europe, and South America. The disease is characterized by high molecular and phenotypic heterogeneity (Smyth et al. 2020). In the last decade, the 5-years overall age-standardized relative survival rate for GC has increased but has remained below 50% (Li et al. 2022). GC patient survival depends greatly on the stage at which they are diagnosed. Patients diagnosed at the localized stage have a 5-years relative survival rate of 77.7%, while those diagnosed at the advanced (distant-stage) have a rate of only 10.2%. Unfortunately, most GC cases are diagnosed at an advanced stage. As a result, early detection, proper therapeutic strategies, and efficient monitoring are crucial in prolonging the survival time for GC patients (Li et al. 2022; Matsuoka and Yashiro 2018; Thrift and El-Serag 2020). However, patients with GC still face the challenge of late detection and poor prognosis, so there is a pressing need to construct a new prognostic and diagnostic model for GC.

Copper homeostasis is closely associated with life-threatening diseases such as cancer, neurodegenerative diseases, and cardiovascular diseases. As a critical component of cell signaling, copper promotes tumor cell proliferation, angiogenesis, and metastasis and therefore contributes to cancer occurrence and progression (Chen et al. 2022). Previous studies revealed that copper promotes tumorigenesis by activating the phosphoinositide 3-kinase (PI3K)-protein kinase B (PKB, also termed AKT) oncogenic signaling pathway through a copper transporter (CTR1) (Guo et al. 2021). Moreover, copper-induced cell death, currently known as cuproptosis, is caused by an ancient mechanism called protein lipoylation, which differs from any other known mechanism of regulated cell death (Tsvetkov et al. 2019; Tsvetkov et al. 2022). In the tricarboxylic acid (TCA) cycle, the increasing lipoylated components serve as a direct binder to copper, which results in an aggregation of lipoylated protein and subsequent downward regulation of Fe-S cluster-containing proteins. Finally, it leads to acute proteotoxic stress and ultimately cell death (Tsvetkov et al. 2022). Given that Cu is integral to human disease, it is reasonable to speculate that cuproptosis may serve as a potential therapeutic target and prognostic indicator. Jian Chen revealed that a novel Cu ionophore, CuS-NiS_2,_ can be successfully applied in the treatment of GC through mitochondria-mediated apoptosis and MLKL/CAPG-mediated necroptosis (Chen et al. 2020). As a newly discovered form of cell death, further research is warranted to identify the key players in cuproptosis and explore the underlying mechanisms. There is a study based on ferredoxin 1 (FDX1), the key regulator of cuproptosis, and its related genes to establish a cuproptosis-related signature to evaluate the prognosis of liver cancer and guide treatment (Zhang et al. 2022a). Determining the molecular features of copper-induced cell death-relevant genes may assist in elucidating the heterogeneity of GC (Wang et al. 2022). We investigated intend to investigate the association of copper-induced cell death-relevant genes with GC prognosis and the tumor microenvironment (TME).

Long noncoding RNAs (lncRNAs) are non‐protein‐coding transcripts with more than 200 nucleotides, comprising intergenic transcripts, enhancer RNAs (eRNAs), and sense or antisense transcripts (Kopp and Mendell 2018; Tan et al. 2021). An emerging regulatory factor, lncRNA interacts with macromolecules such as DNA, RNA, and proteins to exert cellular effects and promote or inhibit tumor progression (Bach and Lee 2018; Beermann et al. 2016; Ma et al. 2018). LncRNAs play an indispensable role in the process of tumorigenesis (Ghafouri-Fard et al. 2020), proliferation (Wang et al. 2020), metastasis (Liu et al. 2021; Luo et al. 2020) drug resistance (Wei et al. 2020) and programmed cell death in cancers (Luo et al. 2022). In general, lncRNAs can participate in tumorigenesis as tumor promoters or tumor suppressors. LncRNA NBR2, a tumor suppressor gene, inhibits autophagy-induced cell proliferation via the ERK and JNK pathways in hepatocellular carcinoma (Sheng et al. 2021). In contrast, PVT1 promoter mutation promotes the process of tumorigenesis and proliferation in cancer (Cho et al. 2018). In addition, lncRNAs are crucial in predicting tumor prognosis. There is a study on crosstalk between lncRNAs and necroptosis, which constructed a prognostic model on the basis of lncRNAs linked to necroptosis in breast cancer (Zhang et al. 2022b). Yangxin exposure in a risk model based on ferroptosis-related lncRNAs is important to the prognosis of hepatocellular carcinoma (Yang et al. 2022). In contrast to the findings in apoptosis, ferroptosis, pyroptosis, and necroptosis, the role of lncRNAs linked to copper-induced cell death in cancer progression and prognosis is still under investigation. Therefore, this study is concerned with the role of copper-induced cell death-related lncRNAs (CRLs) in GC.

As another recently defined mode of cell death, cuproptosis remains largely unknown in GC. In the current research, we aimed to establish a copper-induced cell death-related lncRNA signature (CRLSig) to evaluate and improve the prognosis of GC (Fig. S1). Additionally, we investigated the relationship between the risk score and clinical features, immune cell infiltration, immunotherapy score, and drug sensitivity. Subsequently, the expression levels of CRLs in the serum of 46 healthy people and 66 patients with GC were detected. The relative expression level of lncRNA AC129926.1 in GC cell lines was further verified. Furthermore, colony formation assays, wound healing assays, and transwell assays confirmed the oncogenic effects of lncRNA AC129926.1. This study aimed to provide novel perceptions for personalized immunotherapy, targeted therapy and clinical prediction of prognosis in GC.

## Methods

### Data source and identification of copper cell death-related LncRNAs

High-throughput sequencing data and clinical information of 375 patients with STAD were downloaded from the TCGA public database, and 7 samples with missing clinical follow-up information were deleted. (https://portal.gdc.cancer.gov/repository). The differentially expressed (DE) lncRNAs and mRNAs of gastric cancer (GC) (tumor tissue and normal tissue) were analyzed by using the “edgeR” and “DESeq2” R packages with the criteria of |logFC|≥ 1 and p value < 0.05. Then the intersection of DE RNA obtained by the two methods was obtained. We searched GeneCards with the keyword “copper cell death” (http://www.genecards.org/) and extracted 10 copper cell death-related genes from the Tsvetkov et al. study, which were intersected with copper cell death-related gene sets (Tsvetkov et al. 2022). Finally, a total of 1951 mRNAs were tentatively identified as copper cell death-related genes (CCDRGs). The DE mRNAs overlapping with these 1951 genes were thought to be differentially expressed copper cell death-related genes (DECCDRGs) in GC (484 mRNAs). We used these 484 DECCDRGs to identify 219 copper cell death-related lncRNAs (CRLs) through a screening based on Pearson’s correlation analysis in GC (Pearson ratio > 0.6 and *p* < 0.001).

### Establishment of the risk model

The 368 GC patients were randomly classified into a training cohort (*n* = 184) and a testing cohort (*n* = 184) using the “caret” R package. In the training cohort, univariate Cox regression analysis was used to identify the overall survival (OS)-related DElncRNAs (*p* < 0.05). The least absolute shrinkage selection operator (LASSO) was used to acquire the risk model. Finally, the best prognosis-related lncRNA was determined by multivariate Cox regression analysis to construct the copper cell death-related lncRNA signature (CRLSig) model. The risk score was calculated with the following computational equation: ExpressionlncRNA1 × CoeflncRNA1 + ExpressionlncRNA2 × CoeflncRNA2 + ExpressionlncRNAn × CoeflncRNAn. The patients were separated into high- and low-risk groups with the cutoff point set as the median value of the risk score.

### Validation of the risk model

Kaplan–Meier curves, risk curves, survival state analysis and ROC curves were used to test the CRLSig model. The expression of CRLSig was visualized in a heatmap.

### Prognostic value of the risk model

The independent prognostic value of the risk model was detected by univariate analysis and multivariate analysis. Kaplan–Meier survival analysis was used to analyze the overall survival (OS) of patients with different clinical characteristics. Nomograms can quantitatively predict the prognosis of GC patients. With the CRLsig-based risk score and independent clinical factors, a nomogram of OS and calibration curve for 1, 3 and 5 years were established.

### Functional enrichment analysis

Gene set enrichment analysis (GSEA) software was used to iderntify the difference in enrichment pathways between low- and high-risk cohorts based on the following criteria: |NES|> 1.5, *p* < 0.05 and FDR < 0.05.

### Analysis of the tumor immune microenvironment

Seven algorithms (CIBERSORT- abs, CIBERSORT, EPIC, MCPCOUNTER, QUANTISEQ, TIMER, XCELL) were used to obtain the infiltration of immune cells in different GC samples. The enrichment scores of 13 immune-related pathways and 16 kinds of immune cells in different risk groups were analyzed by single sample gene set enrichment analysis (ssGSEA). The stromal score, immune score and ESTIMATE score (stromal score + immune score) of GC patients were obtained by the “estimating stromal cells and immune cells in malignant tumor tissue based on expression data (ESTIMATE)” algorithm, and then, the scores of different risk groups were compared.

### Assessment of the potential therapeutic drugs

First, the differential expression of 47 immune checkpoints in the high- and low-risk groups was compared. The “prophetic” R package was used to predict the potential drug sensitivity of two risk cohorts of GC patients.

### Determination of the molecular subtypes in GC

Consensus clustering was applied to identify distinct patterns by the k-means method. The number of clusters and their stability were determined by the consensus clustering algorithm using the “ConsensuClusterPlus” package. The R packege Rtsne was used for t-distribution random neighbor embedding (t-SNE) and principal component analysis (PCA).

### Human GC clinical samples

Serum samples of 66 patients with GC and 46 healthy controls were collected from Qilu Hospital of Shandong University from January 2021 to December 2022. This study has been approved by the Medical Ethics Committee of Qilu Hospital of Shandong University (KYLL-202011-223-1).

### Cell culture and overexpression vector transfection

GES-1, AGS and HGC-27 cell lines were cultured in 1640 basic medium with 10% fetal bovine serum (Eisentec), 100 u/ml penicillin and 100 ug/ml streptomycin (Solarbio). The cells were incubated at 37 °C in 5% CO_2_. The medium was changed every other day. The cells were divided into a vector group and an AC129926.1-overexpression group. The GV658-AC129926.1 vector was purchased from GeneChem Co., Ltd (Shanghai, China). 2.5ug of overexpression vector and 5 µl of Lipofectamine 3000 (Invitrogen, USA) were diluted with 125 ul of Opti-MEM.

### Wound scratch assay

GC cells were seeded in 12-well plates, and the wound was scratched manually. The wound healing or cell migration rate was judged by the cell-free area at 0 and 24 h, respectively.

### Colony formation assay

GC cells (1000 per well) were seeded in 6-well plates. After 7 days of culture, the cells were stained with 0.1% crystal violet. Cell clones (> 50 cells) were imaged under a light microscope and quantified manually.

### Transwell migration assay

We measured the migration of GC cells by the Transwell method. We transferred 3*10^4^ cells into the top chamber with serum-free medium, added serum medium into the bottom chamber, and incubated the cells at 37 °C for 24 h. Subsequently, we removed non-invasive cells at the top of the membrane by washing, fixed the migrated cells with 4% paraformaldehyde (PFA), and stained them with 0.1% crystal violet. The total number of cells was counted under a light microscope.

### Isolation of total RNA with quantitative real-time PCR

Total serum RNA was extracted using TRIzol LS Reagent (Invitrogen, Carlsbad, CA). RNA was then reverse transcribed to generate cDNA utilizing an HiScript III RT SuperMix^®^ for qPCR with gDNA Wiper (Vazyme, Nanjing, China). The expression level of AC129926.1 was detected by ChamQ Universal SYBR qPCR Master Mix (Vazyme, Nanjing, China). With GAPDH as the internal reference, the expression of RNA was analyzed and quantified by the 2^−ΔΔCt^ method. The primer sequences are shown in Table S1.

### Statistical analysis

SPSS 25.0 software and GraphPad Prism 9 were used for statistical analysis. Student’s test was used to compare the differences between two or more groups. The chi-square test and Fisher’s exact probability method were used to analyze the correlation between CRLSig and the clinicopathological features of patients with GC. *p* < 0.05 was considered a significant statistical difference. The risk model was constructed by R software (v4.1.3).

## Results

### Identification of the differentially expressed copper cell death-related lncRNAs in gastric cancer patients and the functional enrichment analysis of the risk signature

**Fig. 1:**
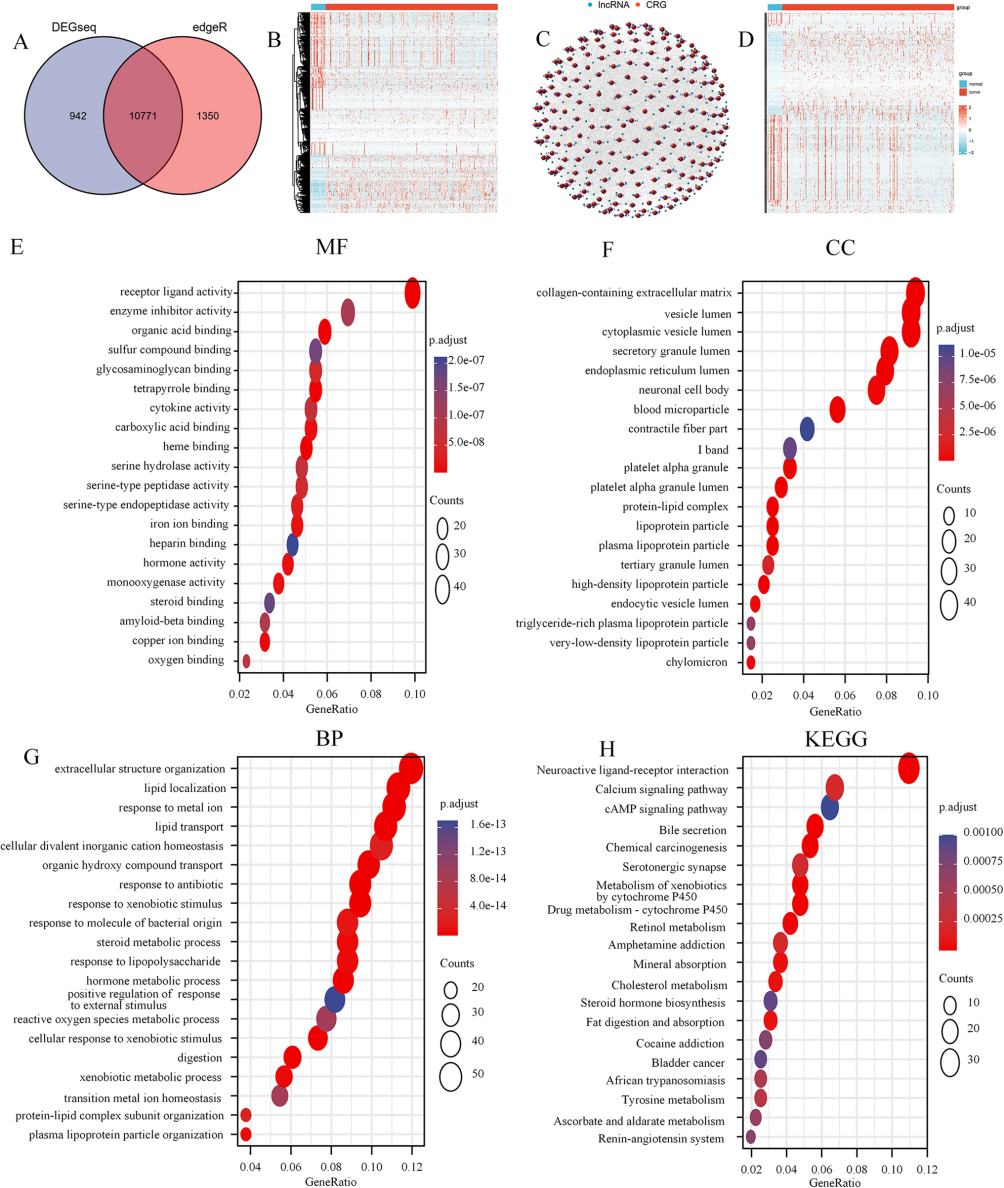
Identification of the differentially expressed CRLs in GC. **A** Identification of the differentially expressed RNAs of GC in the TCGA database through a Venn diagram. **B** Heatmap of the differentially expressed copper cell death related genes. **C** Analysis of crosstalk in the lncRNA-CRG relationship. **D** Heatmap for the differentially expressed CRLs in GC. **E**–**G** GO enrichment analysis of CRGs. **H** KEGG enrichment analysis of CRGs. *CRLs* copper cell death-related lncRNAs, *CRGs* copper cell death related genes

We used DEGseq and edgeR methods to conduct differential expression analysis of mRNA and lncRNA of STAD patients in the TCGA database, and the intersection of the results of the two methods revealed that a total of 10,771 RNAs were differentially expressed (Fig. 1A). There were 484 differentially expressed copper cell death related genes (CRGs) (Fig. 1B). Based on Pearson correlation analysis, we identified 219 differentially expressed CRLs (Fig. 1C, D), which showed the interaction between the CRGs and CRLs. To determine potential biological roles that were correlated with CRGs, we performed a functional enrichment analysis. The MF GO terms indicated that the top three enriched pathways were receptor ligand activity, enzyme inhibitor activity, and organic acid binding (Fig. 1E). The CC GO terms showed that the top three enriched pathways were vesicle lumen, collagen-containing extracellular matrix, and cytoplasmic vesicle lumen (Fig. 1F). The BP terms showed that the top three enriched pathways were extracellular structure organization, lipid localization, and response to metal ions (Fig. 1G). The KEGG pathway enrichment results indicated that CRGs were mainly enriched in the calcium signaling pathway, cAMP signaling pathway, and neuroactive ligand-receptor interaction (Fig. 1H). These results suggest that CRG may be functionally involved in the regulation of cuproptosis-related networks.

### Establishing a 5-copper cell death-related lncRNA risk model for patients with GC.

**Fig. 2:**
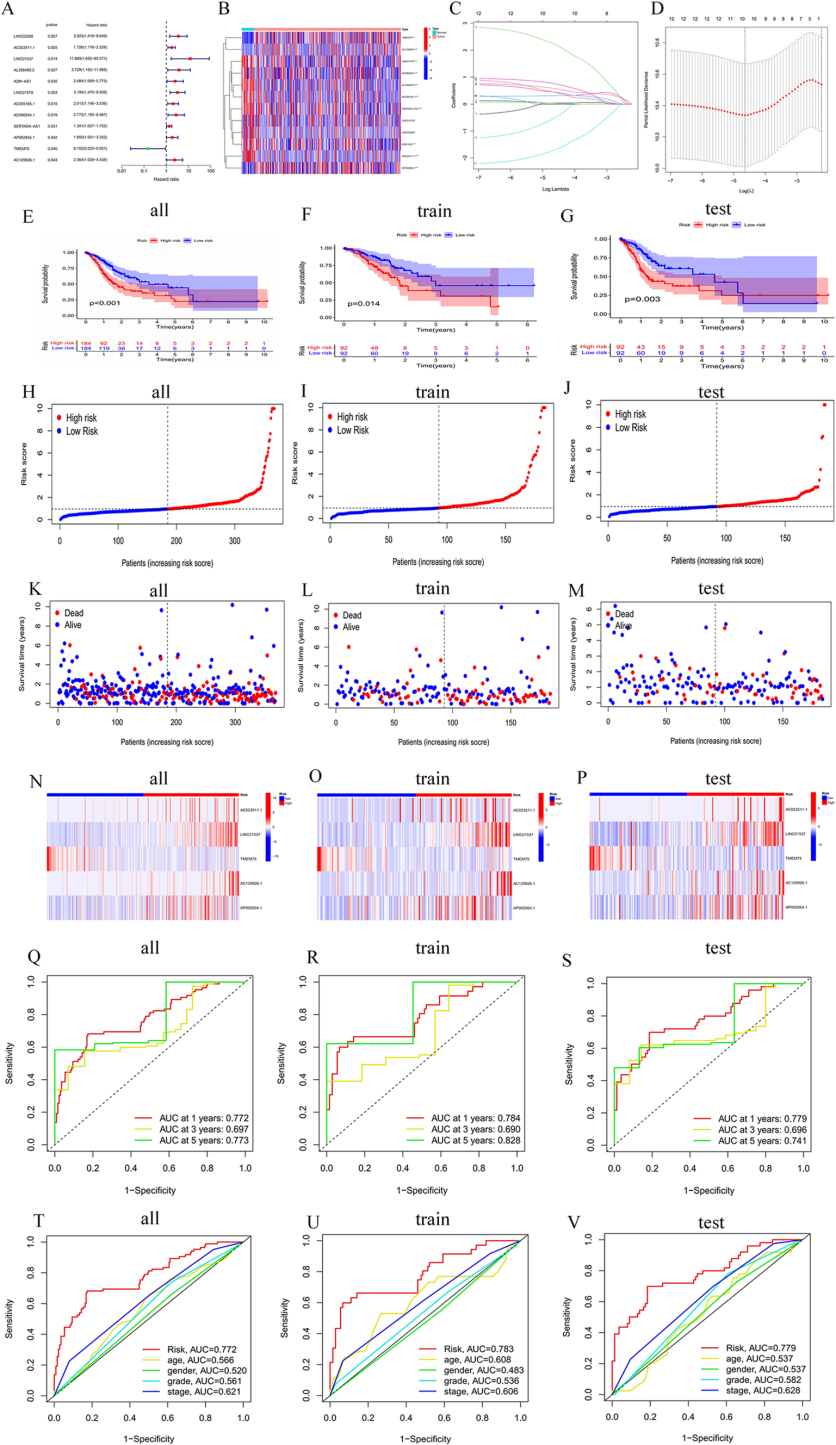
Establishment and verification of the 5 CRLSig risk model in all cohorts, and the training and testing cohorts. **A** The forest plots of the univariate Cox regression analysis between the 12 CRLs and OS of GC. **B** Heatmap of the 12 CRLs in GC. **C** Ten CRLs were selected by the LASSO regression model according to minimum criteria. **D** The coefficient of CRLs was calculated by LASSO regression. **E**–**G** Kaplan–Meier survival curves of the OS of high-risk and low-risk patients in the three cohorts. **H**–**J** The distributions of risk scores in the two risk groups for the three cohorts. **K**–**M** Different patterns of survival status and survival time in the two risk groups for the three cohorts. **N**–**P** Heatmap of the expression of the 5 prognostic lncRNAs in the three cohorts. **Q**–**S** The 1-, 3-, and 5-year ROC curves of the risk model in the three cohorts. **T**–**V** The discriminatory power of the risk model and other clinical factors shown by the ROC curve of OS in the three cohorts

A total of 368 GC samples were randomly divided into a training cohort (*n* = 184) and a testing cohort (*n* = 184). Based on univariate Cox regression analysis, we discovered 12 prognostic copper cell death-related lncRNAs (CRLs) (*p* < 0.05) (Fig. 2A, B). Further Lasso regression was carried out and 10 lncRNAs were screened (Fig. 2C, D). Five CRLs (AC129926.1, AP002954.1, AC023511.1, LINC01537, TMEM75) were screened out by multivariate Cox regression analysis. The risk score of each patient was calculated according to the following risk formula: risk score = AC129926.1 * (0.950) + AP002954.1 * (0.457922243493113) + AC023511.1 * (0.433) + LINC01537 * (3.234) + TMEM75 * (− 1.989). With the median risk score as the cutoff value, 368 cases of GC were divided into high- and low-risk groups. The results showed that the OS of the high-risk group was worse than that of the low-risk group (Fig. 2E–G). The risk score distribution is shown in Fig. 2H–J. More deaths were observed in the high-risk group (Fig. 2K–M). The expression of 5 CRLs in each cohort is shown in Fig. 2N–P. The results showed that the areas under the curve (AUCs) of the entire TCGA cohort at 1-, 3- and 5-years were: 0.772, 0.697 and 0.773, respectively (Fig. 2Q); the AUCs of the training cohort at 1, 3, and 5 years were 0.784, 0.690, and 0.828, respectively (Fig. 2R); and the AUCs of the testing cohort at 1, 3, and 5 years were 0.779, 0.696 and 0.741, respectively (Fig. 2S). In addition, we also found that the risk score was superior to other clinical variables in predicting the prognosis of patients with GC, indicating the superiority of our model in predicting GC survival (Fig. 2T–V). The detailed clinical parameters of the training and testing cohorts are shown in Table S2.

### Independent prognostic value of the copper cell death-related lncRNA signature for patients with GC

**Fig. 3:**
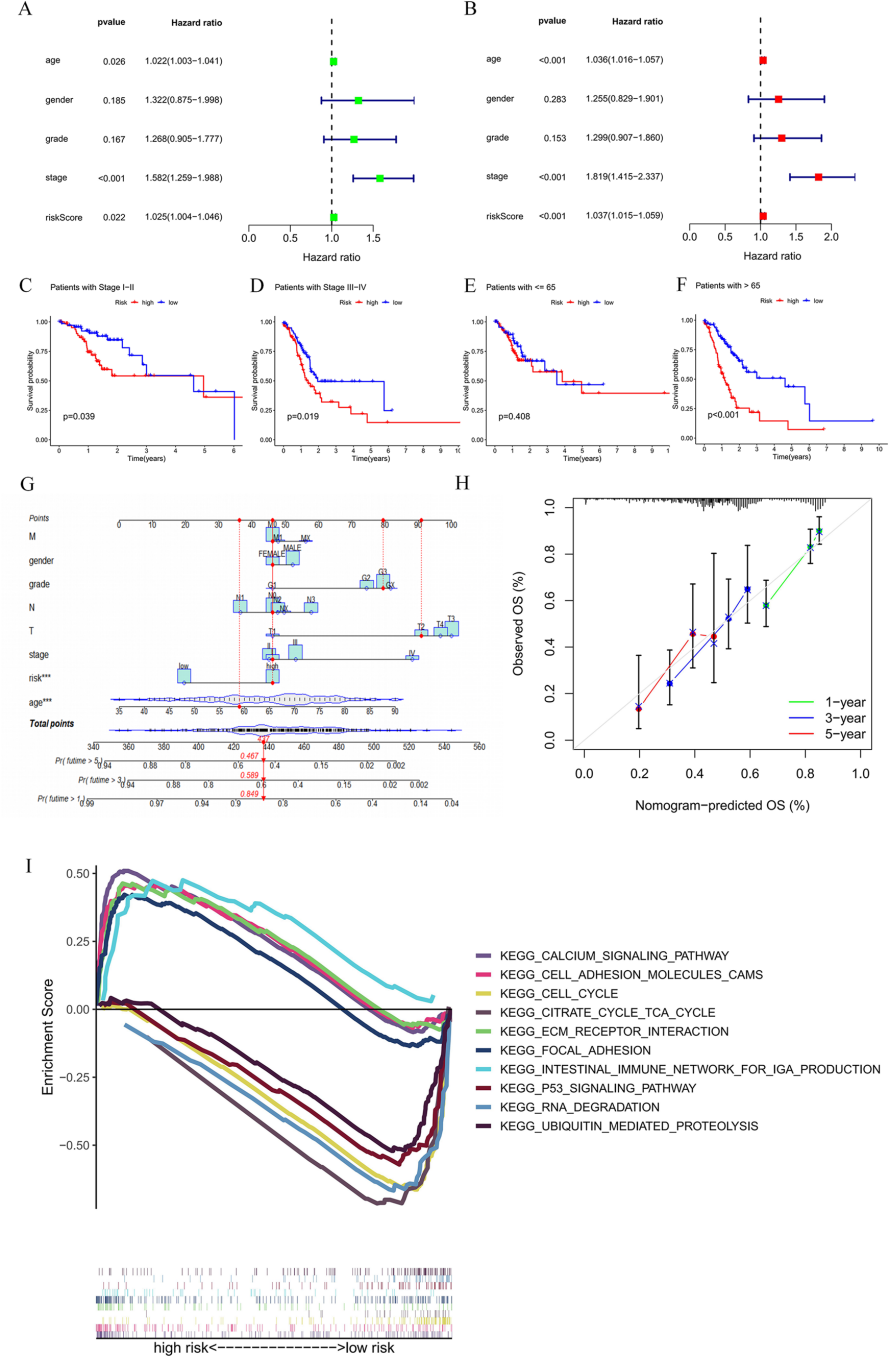
Independent prognostic value of the CRLSig for patients with GC. **A**–**B** Validation of the independence of the CRLSig in OS through the univariate Cox regression analysis (**A**) and multivariate Cox regression analysis (**B**). **C**–**F** The prognostic value was evaluated based on stage (**C**–**D**) and age (**E**–**F**) in the total TCGA cohort. **G** A nomogram was constructed based on the independent prognostic factors to predict the prognosis of GC. **H** The calibration curves for 1-, 3- and 5-year OS plots comparing the actual to predicted values in the TCGA cohort. **I** KEGG pathway analysis for high and low-risk GC patients

Based on the total cohort, a nomogram was constructed to predict the survival risk of GC patients. First, univariate and multivariate Cox regression analyses were used to determine the independent prognostic value of risk scores for CRLSig, age, sex, grade, and stage of patients with GC. The results are shown in Fig. 3A, B, indicating that the risk score of the model is an independent prognostic factor for GC patients (*p* = 0.022, hazard ratiao (HR) = 1.025, 95% CI = 1.004–1.046, Fig. 3A; *p* < 0.001, HR = 1.037, 95% CI = 1.015–1.059, Fig. 3B). We also calculated two other independent prognostic factors: age (*p* < 0.001, HR = 1.036, 95% CI = 1.016–1.057) and stage (*p* < 0.001, HR = 1.819, 95% CI = 1.415–2.237, Fig. 3B). The Kaplan–Meier survival curve showed that OS was significantly better in patients with lower risk scores in stages I–II and stage III–IV and in patients aged > 65 years (Fig. 3C, D, F). There was no significant difference in survival time between high- and low-risk patients when they were 65 years or less (Fig. 3E). In addition, a nomogram of 1-, 3- and 5-year OS was drawn according to age, sex, grade, T stage, N stage, M stage, and risk. The TCGA-RD-A8N2 sample was used as an example to evaluate the nomogram. The patient was 59 years old, female, grade 3, stage I, T2, M0 and N0 and belonged to the high-risk group. The risk scores of patients were 437 and showed a possibility of 84.9%, 58.9% and 46.7% for the OS at 1, 3, and 5 years, respectively (Fig. 3G). Calibration curve analysis showed that the 1-, 3-, and 5-years survival rates observed in the prediction model were in good agreement with the predicted survival rates (Fig. 3H).

### KEGG pathway analysis for high- and low-risk GC patients

To explore the mechanism between the two risk groups, we used GSEA software to find the pathway of KEGG enrichment. Twenty-nine and 89 pathways (all |NES|> 1.5, NOM *p* < 0.05 and FDR < 0.25) were enriched in the high- and low-risk groups, respectively. Five pathways were mapped from the high- and low-risk groups, including the calcium signaling pathway, focal adhesion, ECM receptor interaction, cell adhesion molecules cams, intestinal immune network for IGA production and cell cycle, RNA degradation, citrate cycle TCA cycle, ubiquitin-mediated proteolysis, and p53 signaling pathway, respectively (Fig. 3I).

### Tumor immune microenvironment analysis

**Fig. 4:**
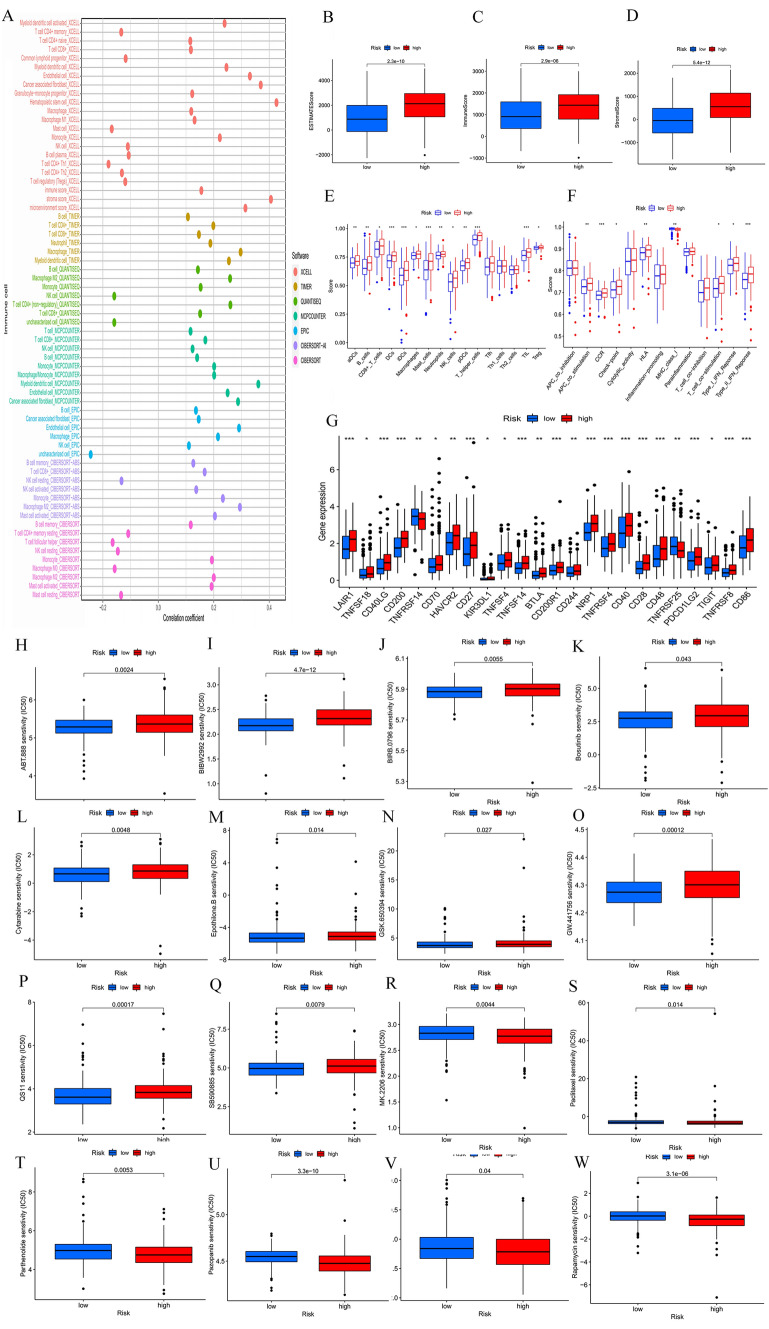
Tumor immune microenvironment analysis and immunotherapy response and drug sensitivity analysis. Tumor immune microenvironment analysis (**A**–**F**). **A** Bubble plot of immune cell infiltration related to the risk model was obtained by different algorithms. **B**–**D** The ESTIMATE, immune, and stromal scores for the tumor microenvironment of GC patients in the high- and low-risk groups. **E**–**F** The ssGSEA scores of immune cells and immune functions in the two risk groups. Immunotherapy response and drug sensitivity analysis. **G** The difference in common immune checkpoint expression in the two risk groups. **H**–**W** The typical antitumor drug chemotherapeutic responses in the low- and high-risk groups. *p < 0.05, **p < 0.01, ***p < 0.001

A bubble chart created using seven different algorithms later revealed that the risk score was negatively correlated with T-cell CD4^+^ Th1, Mast cell, T-cell follicular helper, NK cell, Macrophage M0, Mast cell resting, T-cell CD4^+^ memory, NK cell resting, T-cell CD4^+^ Th2, T-cell regulatory (Tregs), Common lymphoid progenitor, T-cell CD4^+^ memory resting and B-cell plasma, while positively correlated with B cell, NK cell, T-cell CD4^+^ naïve, T cell, B-cell memory, T-cell CD8^+^, Macrophage, Granulocyte-monocyte progenitor, Macrophage M1, NK cell activated, Cancer-associated fibroblast, Monocyte, Neutrophil, Mast cell activated, Monocyte, T-cell CD4^+^, Macrophage M2, Myeloid dendritic cell activated, Myeloid dendritic cell, Endothelial cell and Hematopoietic stem cell (Fig. 4A, Table S3). ESTIMATE analysis showed that all the stromal, immune, and ESTIMATE scores for the tumor microenvironment of patients with GC were higher in the high-risk group (Fig. 4B–D). This finding indicates that the level of immune cells in the tumor microenvironment of the high-risk group is higher. Additionally, ssGSEA was used to compare the immune cell infiltration and immune-related pathways between the low- and high-risk groups. We found that the enrichment scores of 14 kinds of immune cells such as CD8^+^ T-cell, macrophages, NK cells and regulatory T cells (Tregs) in the high-risk group were higher than those in the low-risk group (Fig. 4E). Immune function analysis showed that the activity of seven pathways such as CCR, checkpoint and HLA in the high-risk group was higher than that in the low-risk group, while the activity of MHC class I in the low-risk group was higher than that in the high-risk group (Fig. 4F). All these data demonstrated that the risk model constructed by CRLSig might correlate with the immune response.

### Immunotherapy response and drug sensitivity analysis

We detected the expression levels of immune checkpoint-related genes in the low- and high-risk groups. Interestingly, only TNFRSF14 was significantly downregulated in the high-risk group, while 23 checkpoint-related genes were significantly upregulated: LAIR1, TNFSF18, CD40LG, CD200, TNFRSF14, CD70, HAVCR2, CD27, KIR3DL1, TNFSF4, BTLA, CD200R1, CD244, NRP1, TNFRSF4, CD40, CD28, CD48, TNFRSF25, PDCD1LG2, TIGIT, TNFRSF8, and CD86, which were higher in the high-risk group than in the low-risk group (Fig. 4G). The results showed that most immune checkpoints were more active in high-risk populations. We evaluated the typical antineoplastic drug IC50 value and explored CRLSig to predict the responsiveness of GC patients to antineoplastic drugs. There were 12 higher IC50-value-typical antitumor drugs in the high-risk group. The low-risk groups had 74 higher IC50-value-typical antitumor drugs. The results showed that patients with GC in the low-risk group were more sensitive to ABT.888, BIBW2992, BIRB.0796, bosutinib, cytarabine, epothilone.B, GSK.650394, GW.441756, QS11, and SB590885 (Fig. 4H–Q). In contrast, the high-risk group was more sensitive to paclitaxel, MK-2206, bryostatin-1, parthenolide, pazopanib, and rapamycin (Fig. 4R–W). These findings suggested that our CRLSig could be a valuable predictor of drug sensitivity.

### Identification of the two distinct CRLSig expression subtypes in gastric cancer

We used consensus clustering on the basis of the 5 CRLSig expression levels that came from the risk model. The patients with GC were clustered into two subtypes, namely, C1 (*n* = 87) and C2 (*n* = 281) (Fig. S2A–C). Then, PCA and t-SNE analysis of 5 CRLSig expression levels were clearly divided into two clusters (Fig. S2D, E), and the predefined high- and low-risk groups could also be divided into 2 clusters (Fig. S2F, G). A Sankey diagram was adopted to display the relationships of clusters with their risk types and clusters (Fig. S2H). Survival analysis showed a lower survival probability (Fig. S2I).

### Expression and clinical value of the 5-CRLSig in the serum of GC

**Fig. 5:**
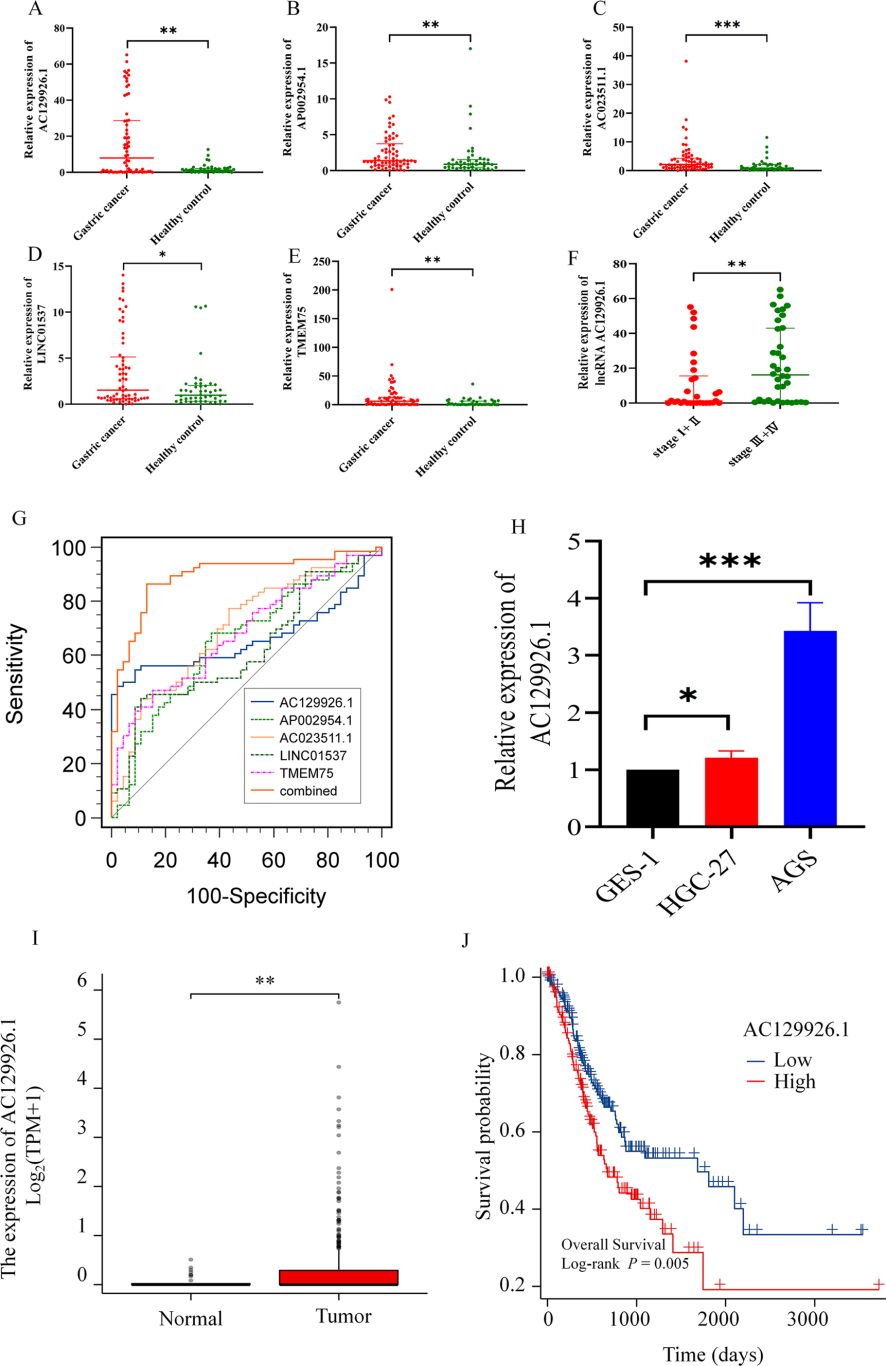
CRLSig levels in the serum of GC patients and healthy controls. **A**–**E** The five CRLSig levels in the serum of GC patients and healthy controls. **F** The expression level of serum lncRNA AC129926.1 in different stages in patients with GC. **G** ROC curve of the combined five CRLs in serum for the diagnosis of GC. **H**–**J** Expression and prognosis of AC129926.1 in GC cell lines and TCGA database. **H** Relative AC129926.1 expression in GES-1, HGC-27 and AGS cells. **I** AC129926.1 expression in TCGA database. **J** Prognosis of AC129926.1 in GC in TCGA database. *p < 0.05, **p < 0.05 and ***p < 0.001

In addition, we sought to explore the clinical utility of these lncRNAs in the risk model. We detected the serum of 46 healthy controls and 66 GC patients to verify the expression of the CRLSig. The results showed that AC129926.1 (*p* < 0.01), AP002954.1 (*p* < 0.01), AC023511.1 (*p* < 0.001), LINC01537 (*p* < 0.05), and TMEM75 (*p* < 0.01) were significantly upregulated in GC serum (Fig. 5A–E). Interestingly, the expression level of serum AC129926 was closely related to the stage of gastric cancer (Fig. 5F). The results showed that serum lncRNA AC129926.1 was much higher in stage III + IV (16.140 (0.812–43.030)) than that in stage I + II (1.136 (0.061–15.580)) (*p* = 0.003). The expression level of lncRNA AC129926.1 in serum of patients with GC increases with the stage. Moreover, we further visualized the ROC curve according to the expression of the CRLs in serum to determine the diagnostic value of the CRLSig in GC. We found that the AUCs of AC129926.1, AP002954.1, AC023511.1, LINC01537, and TMEM75 were 0.662, 0.649, 0.701, 0.622 and 0.681, respectively (Fig. 5G, Table S4). The sensitivity and specificity of these above molecules were 48.48% and 97.83%, 68.18% and 63.04%, 77.27% and 56.52%, 43.94% and 89.13%, 46.97% and 84.78%, respectively. Interestingly, the AUC of the combined 5 CRLs was 0.894 with sensitivity 86.36% and specificity 86.96%.

### Effects of AC129926.1 on the progression of gastric cancer

**Fig. 6:**
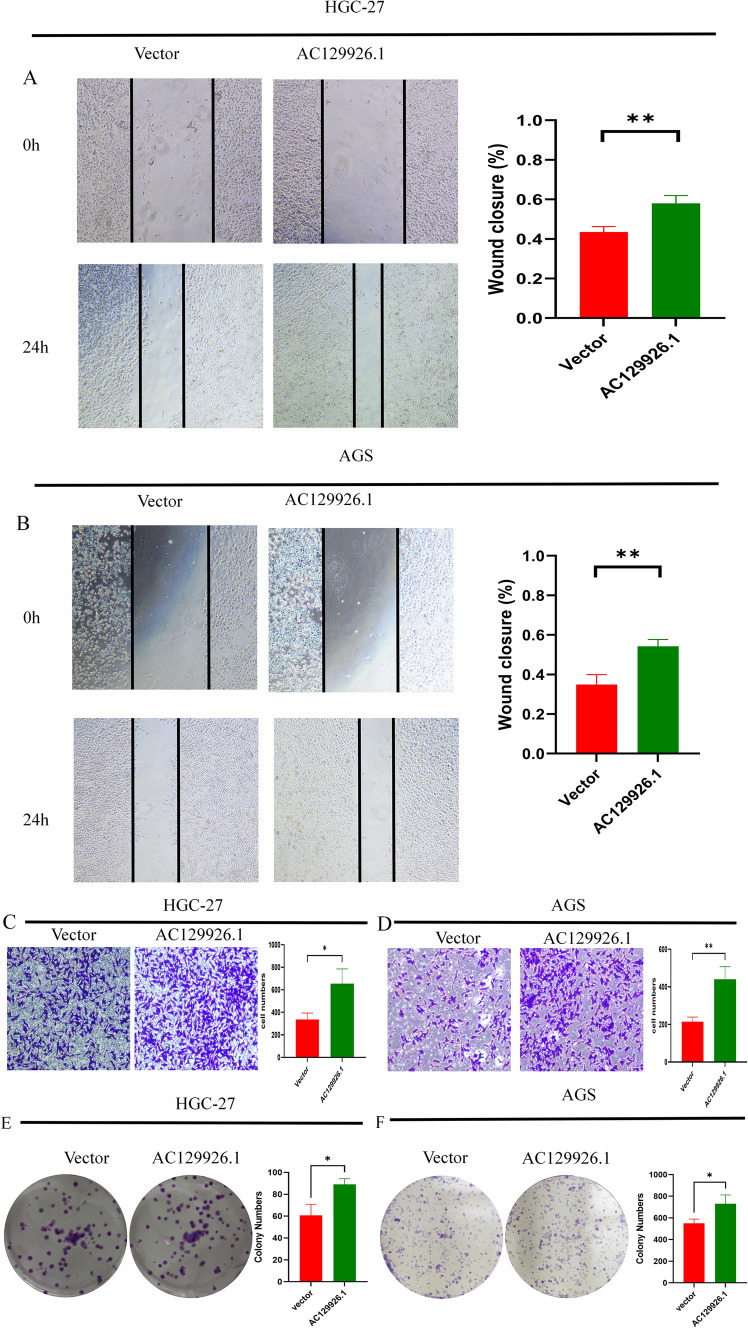
Biological function of AC129926.1 in gastric cancer. **A**, **B** Wound scratch assay results showing that AC129926.1 promotes HGC-27 and AGS cell migration, magnification 100 × . **C**, **D** AC129926.1 significantly promotes the migration of HGC-27 and AGS cells, as detected by Transwell assays. **E**, **F** AC129926.1 enhances the colony formation of HGC-27 and AGS cells in vitro. *p < 0.05, **p < 0.01

Since AC129926.1 was first identified as a significant HR in GC, we chose it to evaluate the potential effects on the occurrence and development of GC. The endogenous expression of AC129926.1 in GC cell lines, including HGC-27 and AGS, and the human normal gastric epithelial cell line GES-1 was measured by RT-PCR. The expression of AC129926.1 was higher in GC cells than in normal gastric cells (Fig. 5H). Given the findings above, we sought to explore the expression and prognostic value of AC129926.1 in primary GC tumors. The TCGA cohort of 375 patients with GC and 32 normal controls was thus analyzed for the AC129926.1 expression. The expression levels of AC129926.1 were significantly higher in GC than in normal tissues (*p* < 0.05) (Fig. 5I). We then aimed to determine whether its deregulation is also related to overall survival (OS) in GC, using the TCGA cohorts. AC129926.1 overexpression was significantly related to a high risk of death in GC (HR = 1.585, 95% CI 1.137–2.208, *p* = 0.0051) (Fig. 5J). Subsequently, AGS and HGC-27 cell lines were transfected with the AC129926.1 overexpression vector to investigate the biological roles of AC129926.1 in GC. Afterward, wound healing assays revealed that AC129926.1 positively regulated the wound healing speed in GC cells (Fig. 6A, B). Transwell assays revealed that AC129926.1 overexpression increased the migration of abilities in HGC-27 and AGS cells (Fig. 6C, D). Colony formation assays suggested that AC129926.1 promotes tumor cell growth in GC cells (Fig. 6E, F).

## Discussion

Gastric cancer (GC) is still a prevalent malignancy worldwide. Although incidence and mortality rates have declined in recent years, GC remains one of the leading causes of cancer death worldwide because of late detection and increased drug resistance (Li et al. 2022; Sung et al. 2021). Further research on the underlying pathogenesis and prognostic factors of GC will be conducive to personalizing the treatment and improving the prognosis of GC. With the proposal of the new concept of cuproptosis, a few studies on the relationship between cuproptosis and the prognosis of various cancers have gradually emerged. There is a reliable, clinically feasible prognostic signature based on Cu-binding proteins (CBP) in GC (Tang et al. 2022). In contrast, the role of copper death-related lncRNAs in the immune landscape of GC remains largely understudied.

In our study, we first obtained and annotated the DE lncRNAs and DEmRNAs in GC from the TCGA database. Moreover, we searched the GeneCards with the keyword “copper cell death” and extracted 10 cuproptosis -related genes from the study of Tsvetkov et al. (Tsvetkov et al. 2022) to screen out the copper cell death-related genes (CCDRGs). Subsequently, the two gene sets were overlapped to obtain 484 differentially expressed CCDRGs in GC. Ultimately, 219 differentially expressed CCDRG-related lncRNAs (CRLs) in GC were obtained by Pearson’s correlation analysis. We also performed functional enrichment analysis of the differentially expressed CCDRGs, which demonstrated that these CCDRGs were primarily responsible for extracellular structure organization, lipid localization, response to metal ions, and the cAMP signaling pathway. Interestingly, these functions or pathways were correlated with the oncogenesis and progression of GC. Transient receptor potential melastatin-2 (TRPM2) ion channels promote GC metastasis by controlling the PTEN/Akt pathway (Almasi et al. 2019). Moreover, Cu2^+^ effectively blocks TRPM2 channels (Zeng et al. 2012). According to these findings, GC development and progression might also be impacted by these differentially expressed CCDRGs. Finally, we constructed a 5-lncRNA prognostic signature based on CRLs, four risk factors (AC023511.1, LINC01537, AC129926.1, AP002954.1) and one protective factor (TMEM75), via the univariate multivariate, LASSO, and multivariate Cox regression analyses. There is a study involving in AC023511.1, a cuproptosis-related lncRNA that may serve as a prognostic predictor of patients with GC, which is consistent with our results (Tu et al. 2022). It has also been reported that LINC01537 promotes GC metastatic development by activating the NF-κB signaling pathway by reducing RIPK4 ubiquitination (Zhong et al. 2022). As an oncogene, TMEM75 promotes colorectal cancer progression relying on the activation of SIM2 (Huang et al. 2022), while its role in GC has not been identified. However, the biological functions of the other two lncRNAs remain largely unknown.

Notably, the 5-CRL prognostic signature could accurately classify the OS outcomes of GC patients into high- and low-risk groups in both the training and testing cohorts. Moreover, the ROC curve indicated that the signature is highly accurate at predicting the OS rate of GC patients across the whole TCGA cohort, training cohort, and testing cohort. In the prediction of GC prognosis, the risk score was superior to other clinical variables, which demonstrates that our model is capable of predicting GC survival with high precision. In addition, univariate analysis and multivariate analysis indicated that the risk score was an independent factor for overall survival. As indicated by the forest plot of the HR value (HR > 1), the risk score represents a problematic factor. Finally, we developed a nomogram to estimate the 1-, 3-, and 5-years survival of patients with GC based on clinical factors and risk scores. The calibration curves demonstrated that the nomogram had excellent predictive capabilities. Overall, compared with traditional clinical factors, the CRL signature has high reliability and validity in predicting the prognosis of GC patients.

Subsequently, GSEA revealed that the TCA cycle is strongly associated with low-risk individuals. Tsvetkov showed that copper-dependent death occurs as a result of direct binding of copper to the TCA cycle (Tsvetkov et al. 2022). TCA can promote the progression of GC through abnormal biological metabolism (Kim et al. 2022). High-risk individuals are closely related to cell adhesion molecules cams, ECM receptor interactions and tumor immunity. Changes in cell adhesion molecules directly affect tumor immune evasion and metastasis (Laubli and Borsig 2019). A previous study demonstrated that the re-expression of poly/oligo-sialylated adhesion molecules on tumor cells can disrupt the interaction between tumor cells and immune effector cells and further lead to pathophysiological immune escape (Jarahian et al. 2021). Additionally, lysyl oxidases (LOXs), copper-dependent monoamine oxidases, are essential for remodeling of the ECM (Lu et al. 2022). ECM plays a role in GC initiation. With the continuous deposition and increasing density of ECM, it directly interacts with receptors on the surface of tumor cells to promote the proliferation, invasion and metastasis of GC cells (Liu et al. 2022). Thus, our signature is likely to provide new insights into the crosstalk between copper-dependent death and TCA in the TME of GC.

Interestingly, the model is also significantly related to immune-related pathways and molecules. According to our study, GC patients with high risk scores exhibited higher stromal, immune, and ESTIMATE scores for the tumor microenvironment. Cancer patients are clinically distinguished by CD8^+^ T-cells and type I interferon (IFN-*α*/*β*) into T-cell-inflamed (CD8 T-cell infiltration and type I interferon marker positivity) and non-T-cell-inflamed patients (lack of both features) (Spranger 2016). The high-risk group was the T-cell-inflamed tumor group based on the enrichment scores and immune function analysis. T-cell-mediated adaptive immunity is thought to play an important role in antitumor immunity (Oya et al. 2020). Importantly, the fundamental cause of immune tolerance and immune escape in tumors is the depletion and functional inhibition of immune cells (Liu et al. 2022). Tumors can evade antitumor immunity not only by enhancing immunosuppressive mechanisms but also by decreasing immunogenicity (Spranger 2016). However, immune checkpoints involve many inhibitory pathways linked to the immune system that are essential for maintaining self-tolerance (Pardoll 2012). Upregulation of PD‐L1 or CTLA‐4 expression inhibits antitumor immune responses in the tumor microenvironment (Oya et al. 2020). T regulatory cells (Tregs) can suppress CD4^+^ and CD8^+^ T-cell-mediated immune responses by suppressing T-cell proliferation, antigen presentation and cytokine production (Oya et al. 2020; Takeuchi and Nishikawa 2016). High FoxP3 (+) Treg infiltration is tightly related to the poor prognosis of most solid tumors (Shang et al. 2015). Tumor-associated macrophages (TAMs) are another key component contributing to the formation of an inhibitory tumor immune microenvironment (TIME) (Shi et al. 2022). The poor outcome of GC is related to the infiltration of IL-10^+^ TAMs, which creates an immunoevasive tumor microenvironment in GC, resulting in the infiltration of regulatory T cells and dysfunction of the CD8^+^ T cells (Zhang et al. 2022c). We found that Tregs and macrophages also presented a high degree of infiltration in the high-risk group. In addition, most immune checkpoints were more active in high-risk populations, which suggests that the high-risk group might have an immunosuppressive microenvironment. Hence, the model has a close connection with the immunosuppressive microenvironment. By presenting antigenic peptides to T cells, major histocompatibility complex (MHC) class I molecules contribute to the cellular immune response (Flutter and Gao 2004). The activity of MHC class I was lower in the high-risk group. In summary, the lncRNA signature may promote GC immune escape through immunosuppressive mechanisms as well as decreased of tumor immunogenicity.

Drug resistance remains a major limiting factor in achieving cures in cancer patients (Vasan et al. 2019). Due to the close relationship between antineoplastic drug efficacy and drug sensitivity, selecting and applying sensitive drugs will significantly improve therapeutic outcomes. For instance, paclitaxel is the basic drug for the first-line treatment of advanced GC (Guo et al. 2015). Sequential paclitaxel combined with bryostatin-1 can significantly improve the response rate of untreated, advanced GC (Ajani et al. 2006). As an Akt inhibitor, MK-2206 enhances carboplatinum paclitaxel efficacy in GC cell lines (Almhanna et al. 2013). Parthenolide is an NF-κB inhibitor that inhibits tumor growth and enhances the response of GC to chemotherapy. Its combination with paclitaxel can significantly prolong the survival of patients (Sohma et al. 2011). VEGF inhibitors have been shown to be beneficial in the second-line treatment of GC. Pazopanib is an oral tyrosine kinase inhibitor that selectively inhibits VEGFR. A randomized phase II trial showed that pazopanib added to chemotherapy was effective but did not show significant improvement (Hogner et al. 2022). Using CRLSig as a predictor of GC patients’ responsiveness to antitumor drugs, we examined the variations in IC50 values between the two groups. GC individuals in the high-risk group showed higher sensitivity to paclitaxel, MK-2206, bryostatin-1, parthenolide, pazopanib, and rapamycin. The low-risk group was more sensitive to ABT.888, BIBW2992, BIRB.0796, bosutinib, cytarabine, epothilone.B, GSK.650394, GW.441756, QS11, and SB590885. According to these findings, our CRLSig may be valuable in predicting drug sensitivity. This method allows for a more detailed analysis of an immunotherapy or targeted therapy’s effectiveness in treating GC patients, and the dose of medication can be adjusted accordingly.

Early staging of patients with GC lacks typical clinical symptoms, and the sensitivity and specificity of traditional biomarkers remain insufficient. LncRNAs are widely and stably present in various body fluids, which provides a novel method for the diagnosis of GC. The AUC of 5-CRLSig in the serum of GC patients was 0.894, and the sensitivity and specificity were 86.36% and 86.96%, respectively. This method had better value for the diagnosis of GC. Previous studies have noted that lncRNA ZFAS1, lncRNA HOXA11-AS, and linc00261 can be used as novel tumor markers for GC screening. The AUCs were 0.85, 0.86, and 0.724, respectively (You et al. 2021; Yu et al. 2017; Zhuo et al. 2022). Thus, the 5 CRLs may contribute to distinguishing GC patients from healthy controls. Among the five lncRNAs in GC, AC129926.1 was found to be a significant HR for the first time. Further survival analysis in TCGA database showed that high relative expression of AC129926.1 was linked to poor prognosis of GC patients. Moreover, AC129926.1 was highly expressed in GC cell lines. Interestingly, AC129926.1 was also highly expressed in the serum of GC patients and was closely related to the stage of GC. As demonstrated by cellular functional experiments, overexpression of AC129926.1 in GC cell lines significantly promoted GC cell proliferation and migration. This finding indicates that AC129926.1 plays an indispensable role in the development of GC. However, its molecular mechanism remains to be further explored.

Overall, the signature of lncRNAs associated with CRLSig may have better predictive efficacy than a single biomarker. Recently, cuproptosis has shown great potential in tumor prognosis prediction. This study preliminarily demonstrated that CRLSig plays a critical role in the progression of GC. It is tightly related to the tumor microenvironment of GC. Importantly, it can serve as a predictor of the prognosis and individual therapeutic strategies of GC. However, this study has certain limitations, including the relatively small sample. Additional studies are needed to further elucidate these findings. In addition, the molecular mechanism of the CRLSig effect associated with GC progression needs to be further explored.

## Conclusion

In summary, a copper cell death-related lncRNA signature (CRLSig) was identified as an innovative prognostic biomarker and potential therapeutic target for patients with GC in our study. The prognostic signature consists of five CRLs, which can accurately evaluate GC patients’ prognosis and characterize their immune landscape. Furthermore, CRLSig provides valuable insight into the diagnosis of GC patients.

## Supplementary Information

Below is the link to the electronic supplementary material.

**Fig. S1** Study design and flowchart of this study. (TIF 840 KB)

**Fig. S2** Identification of the five distinct CRL expression subtypes in GC. **A** Consensus clustering heatmap shows the optimal classification of GC samples with *K* = 2. **B**, **C** Consensus clustering cumulative distribution function fork= 2–9. **D**–**G** PCA and t-SNE analysis of the expression of 5 CRLs. **H** Sankey diagram of clusters with their risk types. **I** Kaplan–Meier survival curves of overall survival (OS) in the two clusters (TIF 989 KB)

**Table S1** Primer sequences (XLSX 10 KB)

**Table S2** Clinical parameters for the training and testing cohorts (XLSX 10 KB)

**Table S3** The correlation of the expression of the CRL signature and the infiltration of immune cells (XLSX 12 KB)

**Table S4** The predictive value of AC023511.1, LINC01537, TMEM75, AC129926.1, and AP002954.1 in gastric cancer (XLSX 10 KB)

## Abbreviations

GC: Gastric cancer
LncRNA: Long noncoding RNA
CRL: Copper cell death-related lncRNA
CRG: Copper cell death-related genes
TCGA: The Cancer Genome Atlas
OS: Overall survival
CRLSig: Copper cell death-related lncRNA signature
TME: Tumor microenvironment
ROC: Receiver operating characteristic
AUC: Area under the curve
IC50: Half-maximal inhibitory concentrations
LASSO: Least absolute shrinkage selection operator
TAM: Tumor-associated macrophage
