# Supplementary figures and images for: A novel copper-induced cell death-related lncRNA prognostic signature associated with immune infiltration and clinical value in gastric cancer
Source: J Cancer Res Clin Oncol. 2023 Jun 8;149(12):10543–59. doi: 10.1007/s00432-023-04916-7 (PMC10423106; doi:10.1007/s00432-023-04916-7)

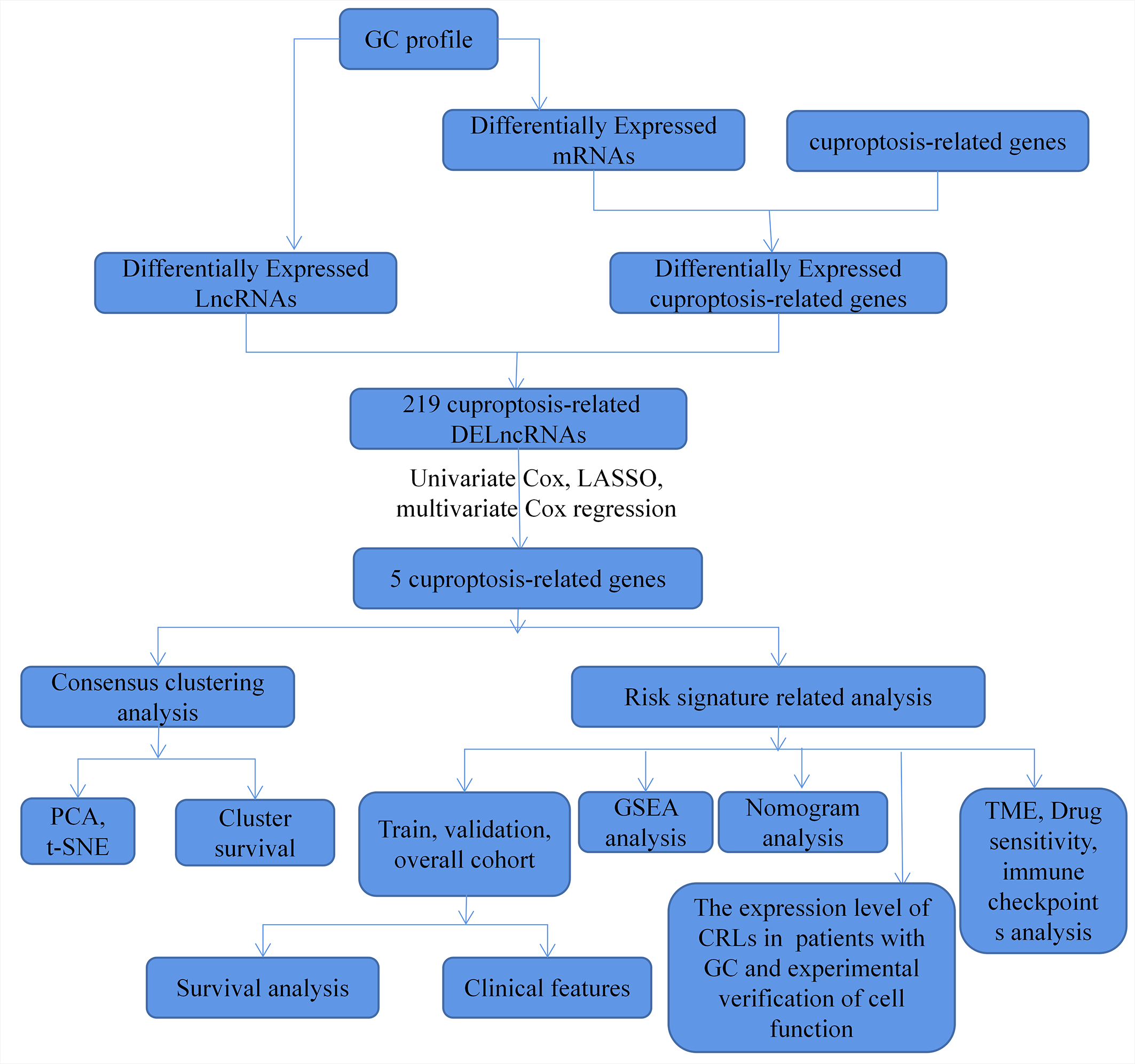

Supplement: Supplementary file 1 — Fig. S1 Study design and flowchart of this study. (TIF 840 KB) [file 432_2023_4916_MOESM1_ESM.tif]

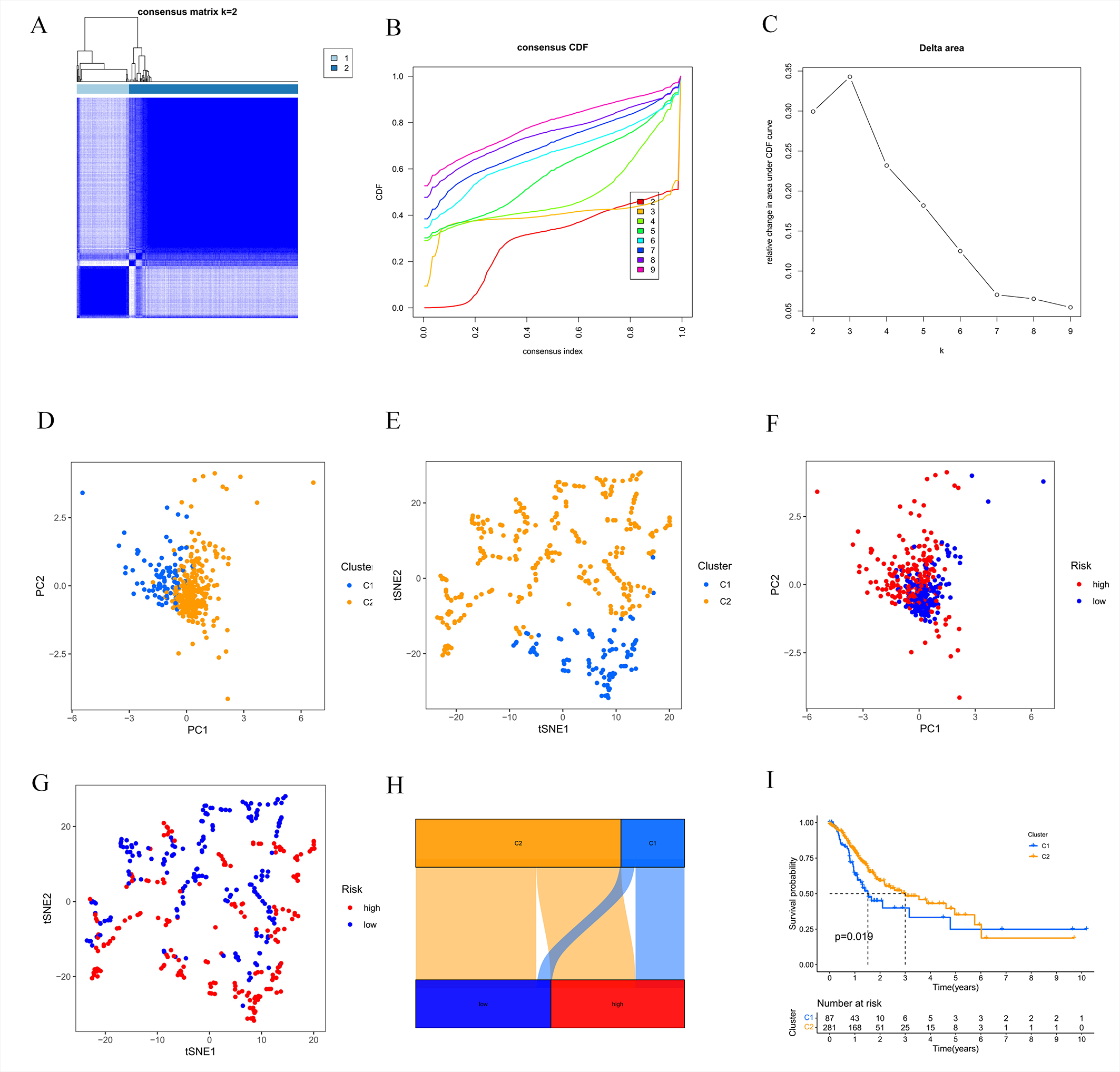

Supplement: Supplementary file 2 — Fig. S2 Identification of the five distinct CRL expression subtypes in GC. A Consensus clustering heatmap shows the optimal classification of GC samples with K = 2. B, C Consensus clustering cumulative distribution function fork= 2–9. D–G PCA and t-SNE analysis of the expression of 5 CRLs. H Sankey diagram of clusters with their risk types. I Kaplan–Meier survival curves of overall survival (OS) in the two clusters (TIF 989 KB) [file 432_2023_4916_MOESM2_ESM.tif]
